# Genotyping of *Salmonella* strains isolated from ducks, their rearing and processing environments in Penang, Malaysia, using RAPD

**DOI:** 10.1007/s13205-013-0115-7

**Published:** 2013-01-18

**Authors:** Frederick Adzitey, Gulam Rusul Rahmat Ali, Nurul Huda, Rosma Ahmad

**Affiliations:** 1 Food Technology Division, School of Industrial Technology, Universiti Sains Malaysia, 11800 Pulau Pinang, Malaysia; 2 Animal Science Department, University for Development Studies, Box TL 1882, Tamale, Ghana

**Keywords:** Ducks, Genetic relatedness, *Salmonella* strains, RAPD

## Abstract

*Salmonella* species are important foodborne pathogens that can cause illness and death in humans. The objective of this study was to determine the genetic relatedness of 115 *Salmonella* strains isolated from ducks and their environment using random amplified polymorphic deoxyribonucleic acid (RAPD). The analysis of *Salmonella* strains by RAPD produced DNA fingerprints of different sizes for differentiation purposes, and cluster analysis at a coefficient of 0.85 grouped the *Salmonella* strains into various clusters and singletons. *S.* Typhimurium were grouped into nine clusters and ten singletons, *S.* Hadar were grouped into seven clusters and nine singletons, *S.* Enteritidis were grouped into four clusters and five singletons, *S.* Braenderup were grouped into five clusters and four singletons, *S.* Albany were grouped into two clusters and seven singletons, and *S*. Derby were grouped into two clusters and four singletons at a coefficient of 0.85 with discriminatory index (*D*) ranging from 0.879 to 0.957. With the exception of *S.* Typhimurium strains which were grouped into three major groups (genotypes) by RAPD analysis, the rest were grouped into two major genotypes. RAPD was a useful genotyping tool for determining the genetic relatedness of the duck *Salmonella* strains. Comparison of the genetic relatedness among foodborne pathogens and their sources of isolation are important to trace their source and possibly the source of human infection.

**Electronic supplementary material:**

The online version of this article (doi:10.1007/s13205-013-0115-7) contains supplementary material, which is available to authorized users.

## Introduction

*Salmonella* species together with other foodborne pathogens are a threat to public health. They are responsible for a number of foodborne outbreaks or sporadic cases that have resulted in foodborne illnesses, hospitalizations and/or deaths. In the United States, Scallan et al. (2011) estimated the pathogens to be a leading cause of human illnesses to be 58 % for norovirus and 11 % for nontyphoidal *Salmonella* spp. Hospitalization-related bacterial foodborne illnesses were estimated to be 35 % for nontyphoidal *Salmonella* spp., 26 % for norovirus and 15 % for *Campylobacter* spp., and that of deaths were estimated to be 28 % for nontyphoidal *Salmonella* spp., 24 % for *T. gondii* and 19 % for *L. monocytogenes* (Scallan et al. 2011). In the UK, 70,298, 9,685 and 176 cases of human campylobacteriosis, salmonellosis and listeriosis, respectively, were reported in 2010 (Defra 2010). In Malaysia, it is cumbersome to estimate the rate of salmonellosis due to inadequately detailed and well-collated epidemiological data from researchers, the veterinary and public health sectors.

*Salmonellae* are widely distributed in food animals, pets, plants, the environment and many other samples (Adams and Moss 2008; Adzitey et al. 2010, 2011; Defra 2010; Frederick and Huda 2011; Abley et al. 2012). The consumption of foods contaminated with foodborne pathogens is responsible for human infections, while contact with surfaces contaminated with foodborne pathogens poses and increases the risk of human infections. Ducks, duck eggs and duck environmental samples in recent times have been reported as very important sources of *Salmonella* (Pan et al. 2010; Adzitey et al. 2012a, b); and contact with ducklings or the consumption of duck eggs, meats or products contaminated with *Salmonella* have been associated with salmonellosis, hospitalization or death of affected persons (Merritt and Herlihy 2003; Noble et al. 2012).

Effective surveillance of *Salmonella* spp. and their outbreak investigations largely depend on efficient isolation, detection and typing methods. A wide range of molecular typing methods have been used to study the genetic relatedness or diversity of bacterial pathogens and even plants (Wassenaar and Newell 2000; Elmeer et al. 2011; Adzitey et al. 2012c, d; Rezk et al. 2012; Tripathi et al. 2012). Genotyping methods such as pulsed field gel electrophoresis (PFGE), multilocus sequence typing (MLST), random amplified polymorphic deoxyribonucleic acid (RAPD), enterobacterial repetitive intergenic consensus (ERIC) and repetitive extragenic palindromic (REP), are among those routinely used to type *Salmonellae* to study their genetic diversity or relatedness (Chansiripornachai et al. 2000; Khoodoo et al. 2002; Lim et al. 2005; Albufera et al. 2009; Smith et al. 2011; Noble et al. 2012). However, published data on the use of RAPD analysis to determine the genetic relatedness of foodborne bacterial pathogens isolated from ducks are rare.

The objective of this study was therefore to ascertain the genetic relatedness of 115 *Salmonella* strains isolated from ducks, and their rearing and processing environments in Penang, Malaysia.

## Materials and methods

### Bacterial strains

A total of 115 *Salmonella* serovars comprising of 37 *S.* Typhimurium, 26 *S.* Hadar, 15 *S.* Enteritidis, 15 *S.* Braenderup, 14 *S.* Albany, and 8 *S.* Derby, isolated from ducks and their environmental sources in Penang, Malaysia, between 2009 and 2010 were used for this study (Adzitey et al. 2012a). *Salmonella* serovars were isolated from duck faeces (*n* = 39), intestines (*n* = 25), cloaca swab (*n* = 14), soil (*n* = 14), wash water (*n* = 8), pond water (4), feed (*n* = 2), drinking water (*n* = 2), carcass rinse (*n* = 3), transport crate swab (*n* = 2), floor swab (*n* = 2) and table swab (*n* = 1) (Adzitey et al. 2012a).

### DNA extraction

A single colony of pure *Salmonella* was inoculated into 10 ml Trypticase-Soy Broth (Merck, Germany) and incubated at a temperature of 37 °C overnight. One millilitre of the overnight culture was centrifuged for 2 min at 14,000×*g*. Pelleted bacterial cells were subjected to DNA extraction using Wizard^®^ Genomic DNA Purification Kit by following the manufacturer’s instructions available at http://www.promega.com/~/media/Files/Resources/Protocols/Technical%20Manuals/0/Wizard%20Genomic%20DNA%20Purification%20Kit%20Protocol.pdf.

### RAPD analysis of duck *Salmonella* isolates

The C-05 (10-mer) primer 5′-GATGACCGCC-3′ was selected for RAPD-PCR after a panel of eight random primers (designed and manufactured by 1st BASE) had been screened. The PCR was performed in a 25 μl volume containing 12.5 μl GoTaq mastermix (M5132, Promega, USA), 6.25 μl nuclease-free water, 2.5 μl 25 mM MgCl_2_, 2.5 μl template DNA (10 μM concentration) and 1.25 μl primer (5 μM concentration). Amplification was performed with the following PCR conditions: initial denaturation at 95 °C for 2 min, followed by 35 cycles at 95 °C for 30 s, 45 °C for 30 s, and 72 °C for 1 min; terminating at 72 °C for 7 min.

All amplifications were performed using Biometra^®^ Tprofessional thermocycler, Germany. Amplicons (10 μl) were stained with EZ-Vision^®^ One DNA Dye (2 μl), loaded on a 1.5 % agarose gel and electrophoresed at 90 V for 1 h 30 min. VC 1 kb and VC 100 bp DNA ladders (Vivantis) were used as the molecular weight marker and the amplicons were visualized under UV transilluminator gel imaging system (Bio-Rad Gel Imaging System).

### Cluster analysis and calculation of discriminatory index

Cluster analysis and calculation of discriminatory index was done individually for the various *Salmonella* serovars. DNA fingerprint positions were determined as described by Adzitey et al. (2012c). Clustering was defined at a coefficient of 0.85. *Salmonella* serovars not belonging to any particular cluster were referred to as singletons (single isolates). Discriminatory index (*D* value) was calculated according to Hunter and Gaston (1988) based on the number of clusters and singletons identified.

## Results and discussion

**Fig. 1:**
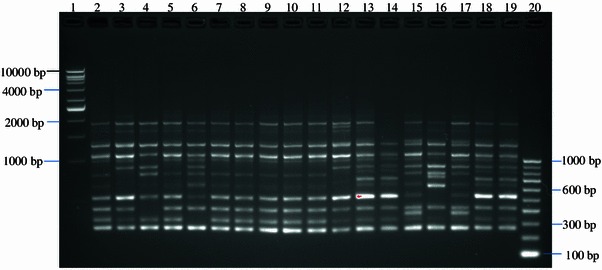
Representative RAPD agarose gel showing DNA fingerprints of *Salmonella* strains.*Lane 1*, 1 kb DNA ladder, Vivantis;*lanes 2–19*, *Salmonella* strains isolated from ducks and their environmental samples;*lane 20*, 100 bp DNA ladder, Vivantis

**Fig. 2:**
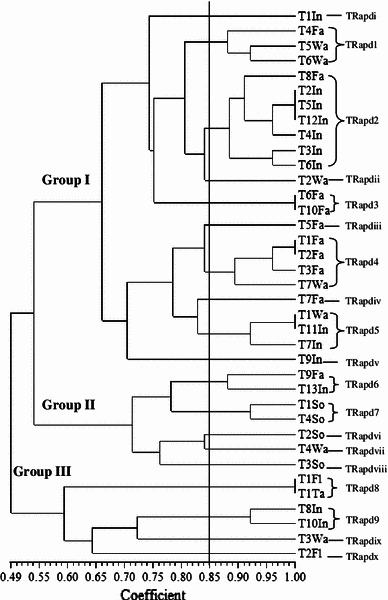
Dendrogram showing the genetic relatedness of *S.* Typhimurium isolated from ducks and their environmental sources performed by RAPD-PCR. TRapd1–TRapd9, *S.* Typhimurium cluster 1–9; TRapdi–TRapdx, *S.* Typhimurium singleton i–x; In, intestines; Fa, faeces; Wa, wash water; So, soil; Fl, floor swab; Ta, table swab

**Fig. 3:**
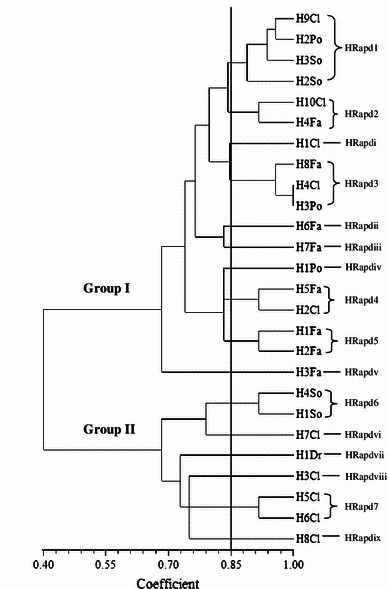
Dendrogram showing the genetic relatedness of *S.* Hadar isolated from ducks and their environmental sources performed by RAPD-PCR. HRapd1–HRapd7, *S.* Hadar cluster 1–7; HRapdi–HRapdix, *S.* Hadar singleton i–ix; Cl, cloaca swab; Po, pond water; So, soil; Fa, faeces; Dr, drinking water

**Fig. 4:**
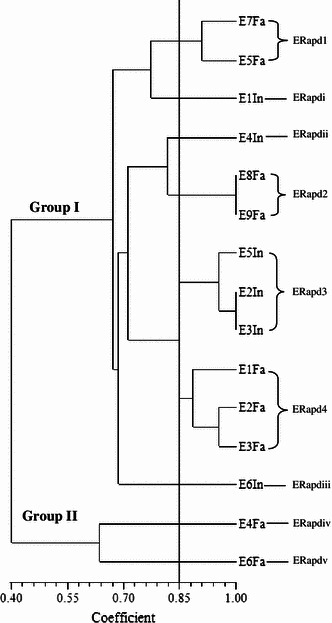
Dendrogram showing the genetic relatedness of *S.* Enteritidis isolated from ducks and their environmental sources performed by RAPD-PCR. ERapd1–ERapd4, *S.* Enteritidis cluster 1–4; ERapdi–ERapdv, *S.* Enteritidis singleton i–v; Fa, faeces; In, intestines

**Fig. 5:**
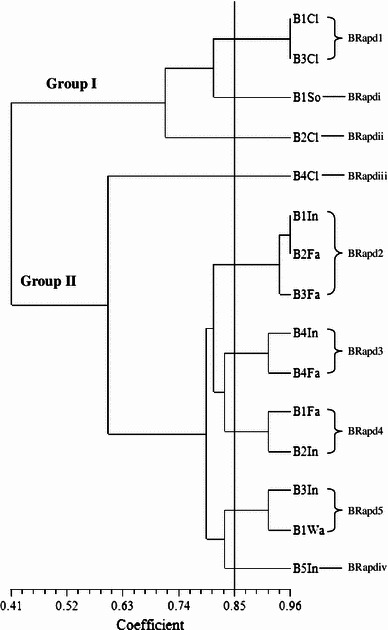
Dendrogram showing the genetic relatedness of *S.* Braenderup isolated from ducks and their environmental sources performed by RAPD-PCR. BRapd1–BRapd5, *S.* Braenderup cluster 1–5; BRapdi–BRapdiv, *S.* Braenderup singleton i–iv; Cl, cloaca swab; So, soil; In, intestines; Fa, faeces; Wa, wash water

**Fig. 6:**
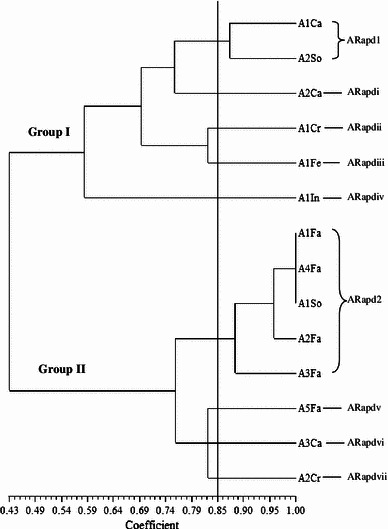
Dendrogram showing the genetic relatedness of *S.* Albany isolated from ducks and their environmental sources performed by RAPD-PCR. ARapd1–ARapd2, *S.* Albany cluster 1–2; ARapdi–ARapdvii, *S.* Albany singleton i–vii; Ca, carcass rinse; So, soil; Cr, crate swab; Cl, cloaca swab; Fe, feed; In, intestines; Fa, faeces

Random amplified polymorphic deoxyribonucleic acid was used to analyse 115 *Salmonellae* isolated from ducks in Penang, Malaysia. RAPD analysis of the *Salmonella* strains produced DNA bands for differentiation purposes. Figure 1 is a representative RAPD PCR agarose gel showing DNA fingerprints of *Salmonella* strains. The reproducibility of the RAPD PCR was verified and confirmed by running the same experiment twice, and the results of both experiments were the same. DNA bands were scored as presence (a score of 1) or absence (a score of 0), and dendrograms (Figs. 1, 2, 3, 4, 5, 6) were constructed from these scores based on simple matching coefficient and Unweighted Pair-Group Arithmetic Average Clustering (UPGMA) using NTSYSpc Version 2.2 computer software. Dendrograms were constructed separately for the various *Salmonella* serovars, and thus, *S.* Typhimurium, *S.* Hadar, *S.* Enteritidis, *S.* Braenderup, *S.* Albany, and *S.* Derby. Clustering was defined at a coefficient of 0.85, and discriminatory index (*D* value) calculated according to Hunter and Gaston (1988) based on the number of clusters and singletons (single isolates). RAPD analysis and clustering of the *Salmonella* strains at a coefficient of 0.85 produced nine clusters and ten singletons for *S.* Typhimurium at a *D* value of 0.943, seven clusters and nine singletons for *S.* Hadar at a *D* value of 0.957, four clusters and five singletons for *S.* Enteritidis at a *D* value of 0.924, five clusters and four singletons for *S.* Braenderup at a *D* value of 0.933, two clusters and seven singletons for *S.* Albany at a *D* value of 0.879, and two clusters and seven singletons for *S.* Derby at a *D* value of 0.929.

**Fig. 7:**
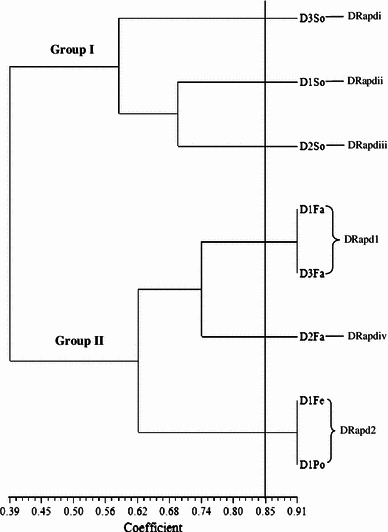
Dendrogram showing the genetic relatedness of *S.* Derby isolated from ducks and their environmental sources performed by RAPD-PCR. DRapd1–DRapd2, *S.* Derby cluster 1–2; DRapdi–DRapdiv, *S.* Derby singleton i–iv; So, soil; Fa, faeces, Fe, feed; In, intestines; Po, pond water

Clusters consisted of two or more *Salmonella* strains and include *S.* Typhimurium cluster 1 (TRapd1), *S*. Hadar cluster 1 (HRapd1), *S.* Enteritidis cluster 1 (ERapd1), *S.* Braenderup cluster 1 (BRapd1), S. Albany cluster 1 (ARapd1) and *S*. Derby cluster 1 (DRapd1), etc. (Figs. 2, 3, 4, 5, 6, 7). *Salmonella* strains in the same cluster are genetically more closely related. Singletons were also observed for all groups of *Salmonella* serovars (Figs. 2, 3, 4, 5, 6, 7), e.g., *S.* Typhimurium T1In (TRapdi), *S*. Hadar H1Cl (HRapdi), *S.* Enteritidis E1In (ERapdi), *S.* Braenderup B1So (BRapdi), *S.* Albany A2Ca (ARapdi) and *S*. Derby D3So (DRapdi) and so on. *Salmonella* strains belonging to these groups have more distant relationships to other *Salmonella* strains.

Figures 2, 3, 4, 5, 6 and 7 also show that the various *Salmonella* serovars were generally grouped into two major genotypes, except for *S.* Typhimurium, which was grouped into three major genotypes. This is expected since the *Salmonella* strains were isolated from the same animal species, similar environment and geographical area. Our results strongly suggest that *Salmonella* strains of similar genotypes were circulating within ducks and their environmental samples in Penang, Malaysia, between 2009 and 2010. *Salmonella* serovars in the same cluster but isolated from different sources could suggest possible cross-contamination. Two examples of such clusters are *S*. Typhimurium cluster 5 (SRapd5) which consists of two isolates from intestines and one isolate from wash water (Fig. 2), and *S*. Albany cluster 2 (ARapd2) which consists of four isolates from faeces and one from soil (Fig. 6). Wash water and soil *Salmonellae* isolates could have been contaminated by intestinal and faecal isolates, respectively. This is because the intestines and faeces of farm animals are known to be primary reservoirs of foodborne pathogens instead of wash water and soil samples (Adams and Moss 2008; Defra 2010; Pan et al. 2010; Adzitey et al. 2012a; EFSA 2012).

Random amplified polymorphic deoxyribonucleic acid analysis depends on the DNA polymorphisms within *Salmonella* strains which are amplified during PCR process to produce DNA fingerprints of different sizes for differentiation purposes. This technique has been used to successfully determine the genetic relatedness and for epidemiological studies of *Salmonella* spp. (Khoodoo et al. 2002; Albufera et al. 2009, Smith et al. 2011). Khoodoo et al. (2002) analysed 19 clinical and 7 local broiler chicken *Salmonella* isolates by RAPD using four arbitrary primers (OPA-10, OPR-03, OPI-06 and OPJ-09) and reported that *Salmonella* isolates from Mauritius were genetically diverse. Albufera et al. (2009) showed that RAPD-PCR analysis of *Salmonella* isolates from human and food sources (fish meat and poultry) generated different profiles for isolates of the same serogroup for differentiation purposes. Smith et al. (2011) screened 61 *Salmonella* spp. (26 clinical, 20 food handlers and 15 animal isolates) by RAPD using four primers and reported that RAPD1 and 2 primers were useful for epidemiological typing of their *Salmonella* spp. The limitation of this study was that only one primer was used after a panel of eight arbitrary primers was screened and the isolates also originated from a very similar source. Nonetheless, our aim was to find out how *Salmonella* species within ducks and their environments relate or diverse from each other genetically using RAPD-PCR.

The RAPD successfully typed all the duck *Salmonella* isolates, showed that *Salmonella* strains of similar genotypes were circulating within ducks/environments in Penang and could also differentiate within some serovariants. This work agrees with findings by Chansiripornachai et al. (2000) who reported that RAPD provided a simple, rapid and cheap typing tool and may be a valuable tool for studying molecular genetic epidemiology of both inter- and intra-serovars of *Salmonella enterica* spp. *enterica.* Hunter and Gaston (1988) reported that a discriminatory index (*D* value) >0.900 is desirable and the typing results can be interpreted with confidence. This study showed that the discriminatory index at a coefficient of 0.85 was mostly >0.900 except *S*. Albany and thus the RAPD PCR was a valuable genotyping tool for the duck *Salmonella* isolates. RAPD can be used together with PFGE, MLST or DNA sequencing to study the genetic diversity or outbreak investigations of *Salmonella* spp. This is because PFGE, MLST and DNA sequencing are generally known to have better discriminatory power and/or reproducibility, which can sometimes be lacking in RAPD (Wassenaar and Newell 2000; Adzitey et al. 2012d). Furthermore, factors such as mutation, horizontal transfer of non-homologous DNA sequences, insertion and deletion of DNA and/or any kind of genetic recombination could contribute to or alter DNA fingerprint patterns and subsequently the genetic diversity of *Salmonella* isolates (Luz et al. 1998; Shabarinath et al. 2007). Lim et al. (2005) compared four molecular typing methods (RAPD, ERIC, ribotyping and single-strand confirmation polymorphism) for the differentiation of 57 strains of *Salmonella* spp. and reported that organisms showing identical fingerprinting patterns are considered to be genetically related, but such relationships depend on the technique applied and no direct correlation existed between the four typing methods used.

## Conclusion

To our knowledge, this is the first extensive report on the use of RAPD PCR to determine the genetic relatedness of *Salmonella* spp. isolated from ducks and their environmental samples in Malaysia. RAPD analysis of the 115 *Salmonella* spp. resulted in strain differentiations and similarities among *Salmonella* strains isolated from the same or different sources. Ducks (eggs, meats and products) and ducklings in recent years have received much attention because of the emergence of *S*. Typhimurium DT8, which has been responsible for a number of foodborne outbreaks. This equally suggests the potential for duck eggs, ducklings and ducks to be important reservoirs for other foodborne pathogens. Knowing the genetic relatedness among *Salmonella* strains is important to know their primary source and possibly their involvement in foodborne infections. The RAPD PCR we adapted was a useful tool for determining the genetic relatedness or diversity of the *Salmonella* strains isolated from ducks and their environmental samples.

## Electronic supplementary material

Below is the link to the electronic supplementary material.

Supplementary material (DOCX 739 kb)
