# Supplementary figures and images for: Genotyping of Salmonella strains isolated from ducks, their rearing and processing environments in Penang, Malaysia, using RAPD
Source: 3 Biotech. 2013 Jan 18;3(6):521–7. doi: 10.1007/s13205-013-0115-7 (PMC3824782; doi:10.1007/s13205-013-0115-7)

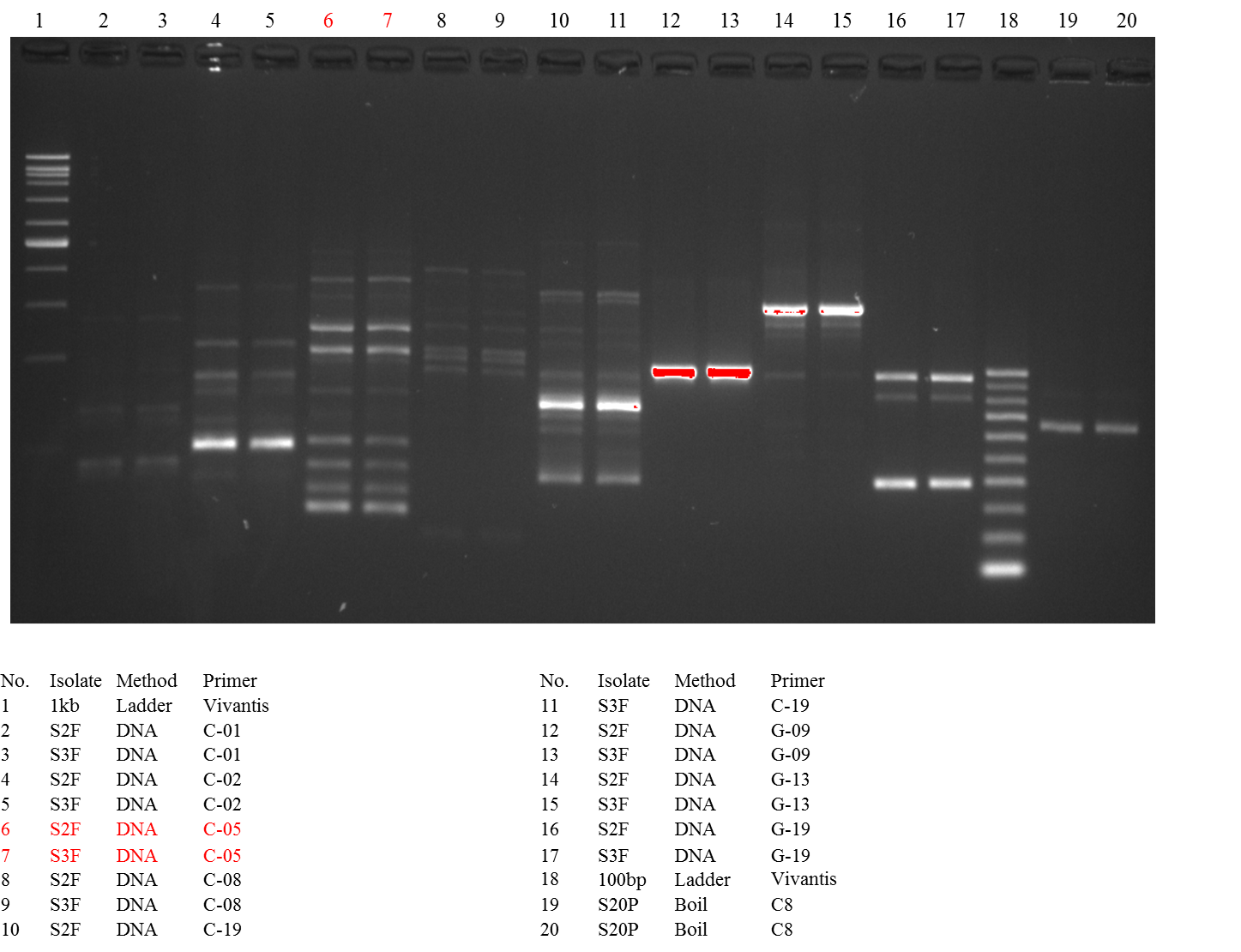


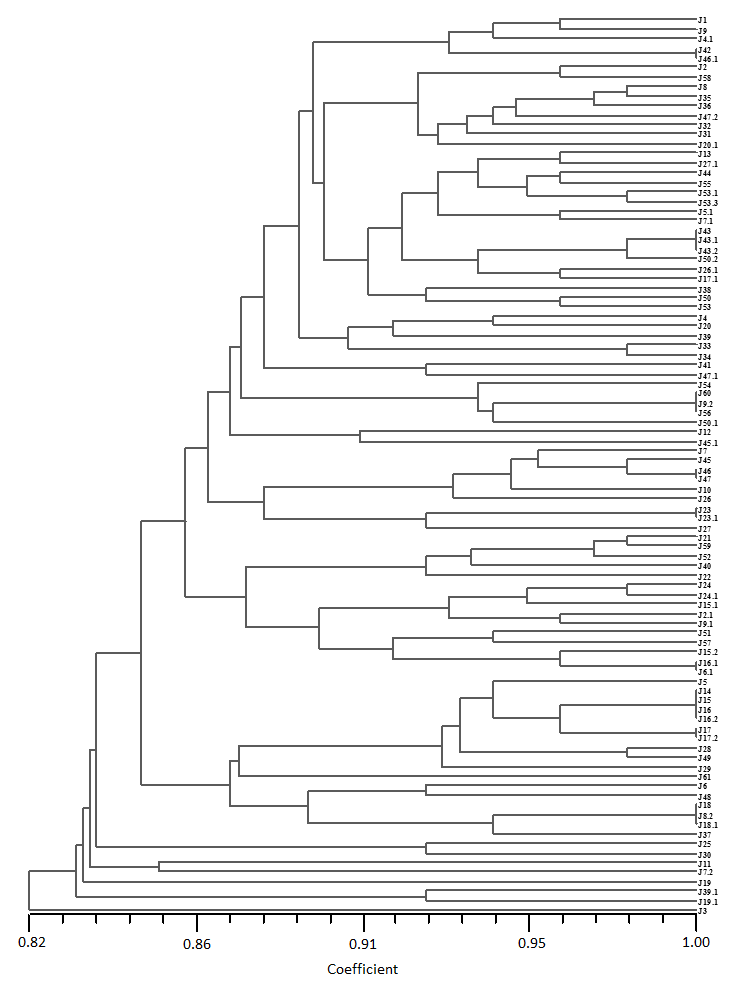

Supplement: Supplementary file 1 — Supplementary material (DOCX 739 kb) [file 13205_2013_115_MOESM1_ESM.docx]
